# Quantitative Analysis of Synthetic Magnetic Resonance Imaging in Alzheimer’s Disease

**DOI:** 10.3389/fnagi.2021.638731

**Published:** 2021-04-12

**Authors:** Baohui Lou, Yuwei Jiang, Chunmei Li, Pu-Yeh Wu, Shuhua Li, Bin Qin, Haibo Chen, Rui Wang, Bing Wu, Min Chen

**Affiliations:** ^1^Department of Radiology, Beijing Hospital, National Center of Gerontology, Institute of Geriatric Medicine, Chinese Academy of Medical Sciences, Beijing, China; ^2^GE Healthcare, Beijing, China; ^3^Department of Neurology, Beijing Hospital, National Center of Gerontology, Institute of Geriatric Medicine, Chinese Academy of Medical Sciences, Beijing, China

**Keywords:** CSF volume, brain volume, multiparametric MRI, cognitive function, Alzheimer’s disease, neurodegenerative disease

## Abstract

**Objectives:** The purpose of this study was to evaluate the feasibility and whether synthetic MRI can benefit diagnosis of Alzheimer’s disease (AD).

**Materials and Methods:** Eighteen patients and eighteen age-matched normal controls (NCs) underwent MR examination. The mini-mental state examination (MMSE) scores were obtained from all patients. The whole brain volumetric characteristics, T1, T2, and proton density (PD) values of different cortical and subcortical regions were obtained. The volumetric characteristics and brain regional relaxation values between AD patients and NCs were compared using independent-samples *t*-test. The correlations between these quantitative parameters and MMSE score were assessed by the Pearson correlation in AD patients.

**Results:** Although the larger volume of cerebrospinal fluid (CSF), lower brain parenchymal volume (BPV), and the ratio of brain parenchymal volume to intracranial volume (BPV/ICV) were found in AD patients compared with NCs, there were no significant differences (*p* > 0.05). T1 values of right insula cortex and T2 values of left hippocampus and right insula cortex were significantly higher in AD patients than in NCs, but T1 values of left caudate showed a reverse trend (*p* < 0.05). As the MMSE score decreased in AD patients, the BPV and BPV/ICV decreased, while the volume of CSF and T1 values of bilateral insula cortex and bilateral hippocampus as well as T2 values of bilateral hippocampus increased (*p* < 0.05).

**Conclusion:** Synthetic MRI not only provides more information to differentiate AD patients from normal controls, but also reflects the disease severity of AD.

## Key Points

1. The brain volume, T1, T2, and PD values can be acquired simultaneously from synthetic MR in a shorter scanning time through one scan.
2. Synthetic MRI can help to distinguish AD patients from normal controls, and it had a relationship with the disease severity of AD.
3. Synthetic MR is a potential approach to be used in AD diagnosis.

## Introduction

Alzheimer’s disease (AD) is a type of progressive neurodegenerative disease, which brings a growing burden to the family and the society in recent years (Alzheimer’s Dementia, 2020). It is challenging to diagnose AD in early diagnosis, due to its occult onset, no specific symptom and image finding. While cerebrospinal fluid (CSF) examination and amyloid positron emission tomography (PET) have been proposed as promising approaches for early detection of AD (Weiner et al., 2015), the wide application of these methods is limited by their high cost, radiation, and invasion. As a non-invasive and non-radiative imaging technique, magnetic resonance imaging (MRI) provides an alternative to explore neuroimaging biomarkers for early detection and diagnosis of AD (Blamire, 2018). Quantitative MRI has been shown to have a potential value in the central nervous system. Specifically, quantitative relaxometry can potentially reflect the changes in tissue characteristics (Deoni, 2010; Callaghan et al., 2014; Knight et al., 2016; Blamire, 2018). However, conventional acquisition of T1 and T2 relaxation times is time-consuming (Bojorquez et al., 2017), and misregistration will be found among different imaging sequences because of the patient motion.

Synthetic MRI is a novel imaging method that simultaneously offers quantitative maps and multiple synthetic contrast-weighted images within a single scan with an acquisition time of several minutes (Warntjes et al., 2008; Gonçalves et al., 2018). T1, T2, and PD values which are the absolute quantification of tissue properties can then be easily measured and compared in the same brain location (Hagiwara et al., 2017b; Gonçalves et al., 2018). A previous study has confirmed that synthetic MRI has good intrascanner repeatability and interscanner reproducibility in the measurements of brain relaxometry (Hagiwara et al., 2019). Furthermore, the volume of gray matter (GM), white matter (WM), CSF, and myelin (MY) content can be acquired by automatic segmentation of the brain tissue based on the relaxation values (Hagiwara et al., 2017b). Using synthetic MRI in central nervous system (CNS) can help to shorten the scan time and reduce discomfort for patients. Therefore, synthetic MRI has its unique superiority in application in CNS.

Synthetic MRI has been extensively applied in CNS diseases such as multiple sclerosis (Granberg et al., 2016; Hagiwara et al., 2017a), vascular diseases (Duchaussoy et al., 2019), and meningitis (Andica et al., 2017). The diagnostic image quality of synthetic contrast-weighted images obtained from synthetic MRI is not inferior compared with that of conventional MR images, and both of them have the similar diagnostic performance in CNS diseases (Tanenbaum et al., 2017). Meanwhile, synthetic MRI can provide additional information including the freedom of synthetic contrasts, volumetric measurements and relaxation times which have a prospect of clinical application (Granberg et al., 2016; Hagiwara et al., 2017a; Duchaussoy et al., 2019). However, to the best of our knowledge, no previous study has examined the clinical value of synthetic MRI in AD diagnosis. Therefore, the aim of this study was to evaluate the feasibility and whether the quantitative information offered by synthetic can benefit the diagnosis of AD.

## Materials and Methods

### Subjects

Eighteen AD patients (five males; age: 68 ± 10 years) and eighteen age-matched (seven males; age: 65 ± 8 years) normal controls were enrolled in this study from July, 2018 to September, 2019. The local institutional review board approved this study. Informed consents were obtained from all participants. The diagnosis of AD was based on the criteria of the National Institute of Neurological and Communicative Disorders and Stroke and the Alzheimer’s Disease and Related Disorders Association (McKhann et al., 1984). All patients underwent the mini-mental state examination (MMSE). The exclusion criteria were as follows: (1) AD patient with other CNS diseases such as brain tumor and contraindications for MRI; (2) normal control with cognitive dysfunction, other CNS disorders and systemic diseases which may cause cognitive impairment.

### MRI Protocol

All subjects underwent MRI examination on a 3.0T MRI scanner (SIGNA Pioneer, GE Healthcare, Milwaukee, WI, United States) and 32-channel head receiver array coil. The scan sequences included a 3D fast spoiled gradient recalled echo (3D-FSPGR) and a synthetic MRI (MAGnetic resonance image Compilation, MAGiC) acquisition. The detail scan parameters were as follows: (1) 3D-FSPGR, FOV = 256 mm × 256 mm, matrix = 256 × 256, slice thickness = 1.0 mm, TR = 6.0 ms, TE = 1.9 ms, flip angle = 11°; (2) MAGiC, FOV = 240 mm × 240 mm, matrix = 192 × 128, bandwidth = 31.25 kHz, echo-train length = 12, slice thickness/gap = 2.0/0 mm, scanning time = 5 min 8 s.

### Post-processing and Measurements

**FIGURE 1:**
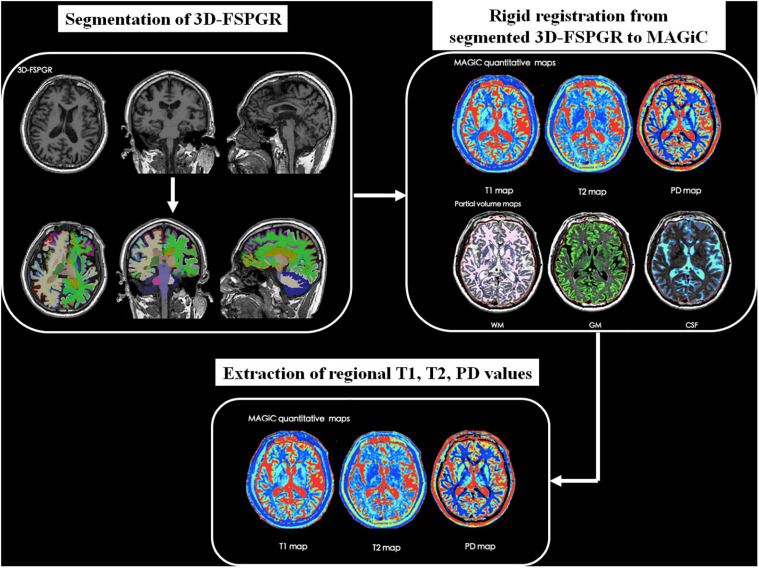
The schematic diagram of post-processing and measurements of synthetic MRI data.

Synthetic MRI data were further processed using SyMRI 8.0 software (SyntheticMR, Linköping, Sweden) to generate relaxation maps. Whole brain volumetric characteristics including the volume of GM, WM, CSF, myelin content, brain parenchymal volume (BPV), intracranial volume (ICV), GM/BPV, WM/BPV, MY/BPV, and BPV/ICV were also acquired by automatic segmentation in the software. To obtain brain regional relaxation values, we first performed parcelation in subject’s native space using FreeSurfer^1^ with Destrieux atlas (Destrieux et al., 2010) based on the 3D-FSPGR images. According to previous studies (Teipel et al., 2015; Chandra et al., 2019), 14 cortical and subcortical regions including bilateral hippocampus, entorhinal cortex, parahippocampal cortex, amygdala, insula, caudate, putamen were chosen as regions of interest (ROIs). After rigid registration between 3D-FSPGR and MAGiC images using MATLAB R2016a (MathWorks, Natick, MA, United States) and SPM12^2^, brain regional T1, T2, and PD values were extracted. Figure 1 shows the schematic diagram of the overall process.

### Statistical Analysis

Statistical analyses were performed using SPSS 25.0 (IBM, Armonk, NY, United States) and MATLAB R2016a. The whole brain volumetric characteristics, brain regional relaxation values were first processed by a linear regression to regress out the effects of age, gender and intracranial volume. Differences of these quantitative parameters between AD patients and normal controls were compared using a two-tailed independent-samples *t-*test. The correlations between quantitative parameters and MMSE scores in AD patients were assessed by the Pearson correlation. Multiple comparisons of brain regional analysis were controlled by false discovery rate (FDR) correction according to 14 selected brain regions. All statistical tests were considered significant with *p* < 0.05.

## Results

### Whole Brain Volumetric Characteristics

Table 1 shows the volumetric characteristics obtained from brain segmentation of synthetic MRI in AD patients and normal controls. Although the AD patients showed larger volume of CSF (*p* = 0.054), lower BPV (*p* = 0.067), and BPV/ICV (*p* = 0.069) compared with normal controls, there were no significant differences (*p* > 0.05).

**TABLE 1:** The parameters obtained from brain segmentation of MAGiC in AD patients and normal controls after regressing out the effects of age, gender, and intracranial volume.

	**AD**	**NC**	***t***	***p***
WM (ml)	32429	33448	0.792	0.434
GM (ml)	52058	54332	1.402	0.170
CSF (ml)	18667	14941	–1.996	0.053
MY (ml)	10420	11311	1.516	0.139
BPV (ml)	85662	88941	1.892	0.067
WM/BPV	0.380.02	0.380.03	–0.484	0.632
GM/BPV	0.590.03	0.600.02	0.629	0.534
MY/BPV	0.130.02	0.130.01	0.390	0.699
BPV/ICV	0.820.05	0.850.03	1.881	0.069

*AD, Alzheimer’s disease; NC, normal control; WM, white matter; GM, gray matter; CSF, cerebrospinal fluid; MY, myelin; BPV, brain parenchymal volume; ICV, intracranial volume.*

### T1, T2 and PD Values of Different Cortical and Subcortical ROIs

T1 values of right insula cortex (1660 ± 125 ms vs. 1553 ± 79 ms, *p* = 0.041) were significantly higher in AD patients than in normal controls. T1 values of left caudate (1431 ± 160 ms vs. 1635 ± 246 ms, *p* = 0.041) were significantly lower in AD patients than in normal controls. T2 values of left hippocampus (149 ± 45 ms vs. 115 ± 23 ms, *p* = 0.046) and right insula cortex (118 ± 15 ms vs. 106 ± 10 ms, *p* = 0.046) were significantly higher in AD patients than in normal controls. PD values of those ROIs showed no significant differences between AD patients and normal controls. All those data were shown in Supplementary Table 1.

### Correlation Between Quantitative Parameters and MMSE Scores in AD Patients

T1 values of bilateral insula cortex and bilateral hippocampus, as well as T2 values of bilateral hippocampus shown in Table 2 increased as the MMSE scores decreased (*p* < 0.05). The whole brain volume characteristics showed different trends. As the MMSE scores decreased, the volume of CSF increased (*p* < 0.05), but the BPV and BPV/ICV decreased (*p* < 0.05).

**TABLE 2:** The correlation between quantitative parameters and MMSE scores in AD patients.

	***r***	***p***
Brain segmentation		
CSF (ml)	–0.664	0.003*
BPV (ml)	0.696	0.001*
BPV/ICV	0.689	0.002*
T1 value (ms)		
Left insula cortex	–0.673	0.010*
Right insula cortex	–0.564	0.041*
Left hippocampus	–0.651	0.012*
Right hippocampus	–0.693	0.010*
T2 value (ms)		
Left hippocampus	–0.758	0.004*
Right hippocampus	–0.631	0.035*

*p < 0.05; AD, Alzheimer’s disease; CSF, cerebrospinal fluid; MMSE, mini-mental state examination; BPV, brain parenchymal volume; ICV, intracranial volume.

## Discussion

### Volumetric Change

Lower BPV and BPV/ICV, as well as higher CSF volume indicated the brain atrophy and an increased ventricular volume. A previous study demonstrated that AD patients had lower total brain volumes and increased size of the lateral ventricles (Kaur et al., 2014). Although our results from brain segmentation of synthetic MRI showed similar larger CSF volume, lower BPV, and BPV/ICV, the differences were not significant which might be attributed to the small sample size. The pathological features of the AD are neurofibrillary tangles (NFTs) and neuritic plaques (NPs) (Querfurth and LaFerla, 2010). Kaur et al. (2014) reported that the brain volumetric change in AD patients had a strong relationship with NFTs, which was also related to the AD induced cognitive decline (Arriagada et al., 1992). The underlying neuropathology leads to global brain atrophy, and these volumetric changes are detectable by synthetic MRI. While we found a smaller volume of GM and WM in AD patient than in normal controls, there was also no significant difference. This result may be explained by the regional and asymmetric brain atrophy. A number of studies have shown that the volumetric reduction was usually found in hippocampus, amygdala, parahippocampal gyrus in AD patients, and this effect may be more severe in the left than in the right hemisphere (Fennema-Notestine et al., 2009; Kaur et al., 2014; Dallaire-Théroux et al., 2017; Ramos Bernardes da Silva Filho et al., 2017; DeVivo et al., 2019). Thus, the regional and asymmetric atrophy may reduce the differences of the whole brain GM and WM volume between AD patients and normal controls. Another reason could be the small sample size. The accumulation of β-amyloid (Aβ) may be one of the critical reasons that lead to AD (Hardy and Selkoe, 2002). Aβ has a relationship with myelin content alterations in the preclinical AD (Dean et al., 2017), and a previous study (Kavroulakis et al., 2018) has shown that myelin content decreased in AD patients. However, while the reduction of MY volume can still be found in AD patient, there was no significant difference as well. The most possible reason is the insufficient samples. Therefore, it is necessary to increase the sample size in the further study.

### Brain Regional Relaxation Values Alteration

Although T1 relaxation time changes may be related to water content, amyloid burden, iron load, and myelin loss in AD patients (El Tannir El Tayara et al., 2006; House et al., 2008; Forster et al., 2013), inconsistent results of T1 alterations were reported both in AD patients and AD transgenic mouse models. Some previous studies have shown decreased T1 values in temporal lobe, parietal lobe, occipital lobe, and basal ganglia of AD patients compared to normal controls (House et al., 2008; Su et al., 2016). We got the similar results in left caudate. However, Kelly et al. (2013) has demonstrated that astrogliosis increased T1 values in AD mouse model. Additionally, the partial volume effects caused by brain atrophy would increase T1 value in the areas of cortex (Blamire, 2018). Therefore, increased T1 values of right insula cortex in AD patients reported by our study will be further studied.

Previous researches demonstrated that T2 relaxation time was associated with tissue water content, iron load, myelin density, and amyloid deposits (Paus et al., 2001; House et al., 2007; Nabuurs et al., 2011). T2 alterations in AD patients were controversial in different studies. Our results suggested that T2 values in AD were significantly higher in left hippocampus compared to normal controls, which is consistent with some previous studies (Wang et al., 2004; Raven et al., 2013; Knight et al., 2019). However, Su et al. (2016) found that T2 values were lower in hippocampus of AD patients. Even though the factors such as tissue water content alterations, iron accumulation, and myelin loss may decrease T2 value (Paus et al., 2001; House et al., 2007; Nabuurs et al., 2011), the partial volume effects caused by atrophy and lacunae would dominate and increase T2 value, due to the extremely high values in CSF (Wang et al., 2004). This pattern can also be found in cortical areas such as insula cortex, temporal cortex, and prefrontal cortex, where have been demonstrated to have volumetric reduction in AD (Dallaire-Théroux et al., 2017; Blamire, 2018). Moreover, a previous meta-analysis (Tang et al., 2018) proved increased T2 value in the hippocampus of AD patients compared to healthy controls.

PD values can provide the information of tissue water content and reflect the structural damage of brain (Gracien et al., 2016). However, the PD value alternation in AD has not yet been well studied. According to our results, although the PD values of right putamen were lower in AD patients than in normal controls, it did not survive after multiple comparison correction. Therefore, future studies about alteration of PD values in AD patients are still needed.

### Association Between Quantitative Parameters and MMSE Scores in AD Patients

The brain atrophy will be more obvious with the progression of AD (Blamire, 2018). In our study, the larger volume of CSF and lower BPV/ICV reflected the brain atrophy and ventricular enlargement. Our results also showed that as the MMSE scores decreased, the volume of CSF increased, while BPV and BPV/ICV decreased, which were consistent with the results reported by Ramos Bernardes da Silva Filho et al. (2017). These results demonstrated that the volumetric characteristics acquired from brain segmentation of synthetic MRI had a correlation with the disease severity of AD. Table 2 showed that T2 values in bilateral hippocampus had a significant negative correlation with MMSE scores, which corroborated with a previous study (Laakso et al., 1996). T1 values in bilateral hippocampus and insula cortex showed the same trend. The reason may be that the volume of hippocampus was obviously decreased as the disease severity of AD developed, and this volumetric reduction will lead to increased T1, T2 values due to partial volume effects. Therefore, there were significant negative correlations between T1, T2 values of hippocampus and MMSE scores. However, Luo et al. (2013) and Su et al. (2016) found the opposite results, and suggested the reasons were the amyloid burden and iron deposition in the brain regions of AD patients. In fact, we also did the comparison of bilateral hippocampus volumes between AD patients and normal controls from the segmentation of FSPGR. The results showed that volumes of bilateral hippocampus in AD patients were significantly lower than those in normal controls (Supplementary Table 2). Hence, we may attribute the higher T1 and T2 values of bilateral hippocampus with lower MMSE scores to the partial volume effect, which may also be the reason of higher T1 values of bilateral insula with lower MMSE scores. In general, the quantitative values obtained simultaneously from synthetic MRI may reflect the disease severity of AD.

There were still several limitations in this study. Firstly, the sample size was relatively constrained in this study. A larger sample size is desirable to further generalize the results observed in this study. Secondly, only single quantitative parameter was investigated in this study. Using the combination of different parameters may have the potential for improving diagnostic accuracy of AD. Thirdly, our research is a cross-sectional study. Further longitudinal study is needed to evaluate the clinical value of synthetic MRI in the disease progression of AD.

In conclusion, synthetic MRI is a novel imaging approach that can offer both quantitative maps and synthetic contrast-weighted images in several minutes. The volume of brain, T1, T2, and PD values can be obtained simultaneously using synthetic MRI. These parameters not only provide more information to differentiate AD patients from normal controls, but may also reflect the disease severity of AD. Overall, synthetic MRI may help to monitor the progression of AD and have a clinical value to explore potential biomarkers for the early detection of AD in the future.

## Data Availability Statement

The raw data supporting the conclusions of this article will be made available by the authors, without undue reservation.

## Ethics Statement

The studies involving human participants were reviewed and approved by the Institutional Human Ethics Review Board of Beijing Hospital. The patients/participants provided their written informed consent to participate in this study.

## Author Contributions

BL and YJ: data analysis and manuscript writing. YJ and CL: participant enrollment and data collection. P-YW and BW: MRI acquisition and MRI processing. SL, BQ, HC, and RW: technique support. MC: idea conceivement, study design, and critical revision of manuscript. All authors contributed to the article and approved the submitted version.

## Conflict of Interest

P-YW and BW were employed by company GE Healthcare. The remaining authors declare that the research was conducted in the absence of any commercial or financial relationships that could be construed as a potential conflict of interest.

## Footnotes

1 https://surfer.nmr.mgh.harvard.edu

2 http://www.fil.ion.ucl.ac.uk/spm/

## Abbreviations

AD: Alzheimer’s disease
A β: β -amyloid
BPV: brain parenchymal volume
CSF: cerebrospinal fluid
CNS: central nervous system
3D-FSPGR: 3D fast spoiled gradient recalled echo
GM: gray matter
ICV: intracranial volume
MRI: magnetic resonance imaging
MY: myelin
MMSE: mini-mental state examination
MAGiC: MAGnetic resonance image Compilation
NFTs: neurofibrillary tangles
NPs: neuritic plaques
PET: positron emission tomography
WM: white matter.
