# Supplementary material for: Quantitative Analysis of Synthetic Magnetic Resonance Imaging in Alzheimer’s Disease
Source: Front Aging Neurosci. 2021 Apr 12;13:638731. doi: 10.3389/fnagi.2021.638731 (PMC8072384; doi:10.3389/fnagi.2021.638731)
Supplement: Supplementary file 1 [file Data_Sheet_1.docx]

Supplementary Table 1. T1, T2 and PD values of 18 cortical and subcortical ROIs.

|  | T1 value (ms; AD vs normal control) | T2 value (ms; AD vs normal control) | PD value (%; AD vs normal control) |
| --- | --- | --- | --- |
| Left hippocampus | 1773±236 | 149±45 | 85±2 |
|  | 1682±140 | 115±23 | 83±1 |
| Right hippocampus | 1780±281 | 160±47 | 85±3 |
|  | 1642±161 | 129±18 | 84±1 |
| Left entorhinal cortex | 1697±265 | 124±33 | 84±3 |
|  | 1643±159 | 114±16 | 83±2 |
| Right entorhinal cortex | 1790±182 | 127±16 | 86±2 |
|  | 1748±180 | 124±13 | 85±2 |
| Left parahippocampal cortex | 1595±202 | 108±25 | 82±2 |
|  | 1490±106 | 99±7 | 81±2 |
| Right parahippocampal cortex | 1597±165 | 112±19 | 80±2 |
|  | 1526±89 | 105±9 | 79±1 |
| Left amygdala | 1385±113 | 101±35 | 81±3 |
|  | 1415±82 | 89±13 | 81±2 |
| Right amygdala | 1436±105 | 110±15 | 82±3 |
|  | 1437±75 | 107±11 | 82±2 |
| Left insula cortex | 1549±132 | 116±8 | 84±2 |
|  | 1456±74 | 111±7 | 83±1 |
| Right insula cortex | 1660±125 | 118±15 | 84±1 |
|  | 1553±79 | 106±10 | 84±1 |
| Left putamen | 1127±62 | 71±8 | 81±2 |
|  | 1147±71 | 73±4 | 81±1 |
| Right putamen | 1078±100 | 71±8 | 79±1 |
|  | 1088±69 | 73±4 | 80±1 |
| Left caudate | 1431±160 | 85±107 | 82±3 |
|  | 1635±246 | 78±54 | 84±3 |
| Right caudate | 1320±165 | 87±31 | 83±3 |
|  | 1460±198 | 106±42 | 84±2 |
| Left posteriorcingulate cortex | 1507±187 | 101±17 | 84±2 |
|  | 1449±133 | 99±11 | 85±1 |
| Right posteriorcingulate cortex | 1518±182 | 89±81 | 84±1 |
|  | 1485±84 | 70±24 | 84±1 |
| Left precuneus | 1419±111 | 95±10 | 82±1 |
|  | 1393±69 | 93±5 | 83±1 |
| Right precuneus | 1464±122 | 93±10 | 82±2 |
|  | 1416±66 | 92±5 | 83±1 |

Supplementary Table 2. Bilateral hippocampus volume obtained from brain segmentation of FSPGR in AD patients and normal controls.

|  | AD | NC | *t* | *p* |
| --- | --- | --- | --- | --- |
| Left hippocampus |  |  |  |  |
| Volume (ml) | 3.18±0.62 | 3.75±0.51 | 3.006 | 0.005* |
|  |  |  |  |  |
| Right hippocampus |  |  |  |  |
| Volume (ml) | 3.36±0.62 | 3.95±0.60 | 2.930 | 0.006* |
